# Near-full-length genome sequences of human norovirus GI.7[P7] and GII.4[P31] strains co-infecting a California patient in 2017

**DOI:** 10.1128/MRA.00617-23

**Published:** 2023-09-29

**Authors:** Zhihui Yang, Mark Mammel, Julia Wolfe

**Affiliations:** 1 Division of Molecular Biology, Office of Applied Research and Safety Assessment, Center for Food Safety and Applied Nutrition, U.S. Food and Drug Administration, Laurel, Maryland, USA; 2 Orange County Health Care Agency, Public Health Laboratory, Santa Ana, California, USA; DOE Joint Genome Institute, Berkeley, California, USA

**Keywords:** human norovirus, whole-genome sequencing, co-infection

## Abstract

Accurate identification of human noroviruses (HuNoVs) from outbreaks or sporadic cases is critical for source tracing and outbreak investigation. Whole-genome sequencing is a powerful tool for the detection, identification, and discrimination of HuNoV strains. We report here the nearly complete genome sequences of GI.7[P7] and GII.4[P31] strains detected in a Californian patient co-infected by both strains in 2017.

## ANNOUNCEMENT

Single-stranded RNA noroviruses (NoVs) (genus Norovirus, family *Caliciviridae*) are the primary cause of epidemic acute gastroenteritis in the US and worldwide (1) (2). NoVs are genetically diverse and classified into 10 genogroups (GI–GX) and further classified into at least 49 genotypes. Strains in genogroups GI, GII, GIV, GVIII, and GIX are human-associated (3). GII (and especially GII.4) has been reported as the dominant genotype in humans and is mainly associated with person-to-person transmission, followed by GI, which is mainly associated with foodborne and waterborne transmission (4) (5, 6). Even though multiple strains of human NoV (HuNoV) could be associated with outbreaks or co-infect individuals (7) (8), detection of multiple HuNoV strains is rarely reported since the current detection methods have limitations on strain identification (9) (6). The development of whole genome sequencing (WGS) technologies makes it possible to detect and identify various co-infecting strains with high accuracy, sensitivity, and specificity (10). Here, we report the successful identification and near-complete genome sequences of GI.7[P7] and GII.4[P31] strains from an individual patient in California in 2017.

A stool sample was collected in Orange County, CA, in 2017. It was detected positive by RT-qPCR (10) for NoV GI and was suspended in 10% (wt/vol) phosphate-buffered saline (PBS). This PBS suspension was filtered (0.65 µm), followed by viral RNA extraction using a QIAampViral RNA Mini Kit (Qiagen). A sequencing library was sequenced on the MiSeq platform (Illumina), generating paired-end 100-bp reads from the RNA using a Revelo Kit (TECAN). Data analysis was performed on the raw data of 6.3 M total reads using our in-house k-mer tool (11) (version vf6) (github.com/mmammel8/kmer_id) to identify viral species as well as sequence-based alignment (against our local database, including representative full-length norovirus sequences downloaded from NCBI) with CLC Genomics Workbench (CLC bio, version 23) (12). One fragment covering the norovirus genome sequence was assembled from 14,710 reads, containing an average coverage of 229 ×. This genome sequence has a length of 7,732 nt and a GC content of 48.6% and was annotated via the GenBank norovirus submission tool in the NCBI Submission Portal. It contains (i) three open reading frames (ORFs): ORF1 (5,397 nt), ORF2 (1,620 nt), and ORF3 (648 nt) and (ii) a 3′-UTR of 86 nt in length. Virus typing demonstrated that this sequence was a GI.7[P7] strain by using the Norovirus Genotyping tool version 1.0 (13) and the CaliciNet lab-based Human Calicivirus Typing Tool Calicivirus typing tool (cdc.gov). In addition to this GI sequence, another fragment covering the norovirus genome sequence was also recovered from 1,123 reads containing a GC content of 49.8% and an average coverage at 19 ×. This genome sequence was 6,960 nt in length, containing three open reading frames: ORF1 (4,551 nt), ORF2 (1,623 nt), and ORF3 (807 nt). Using the same genotyping tools as above, this sequence was typed as a GII.4 Sydney_2012[P31] strain and was not detected by RT-qPCR. These results demonstrate that WGS could potentially be used as a confirmatory method for HuNoV detection and that it is also a powerful tool to provide genomic information missed by other assays.

## Data Availability

The genome sequences of these GI.7[P7] and GII.4 Sydney_2012[P31] strains have been established in GenBank under accession numbers OR065043 and OR086127, respectively. The raw sequence data have been deposited in SRA under the Virotrakr, accession number SAMN35429855.
